# Non-Cooperative SAR Automatic Target Recognition Based on Scattering Centers Models

**DOI:** 10.3390/s22031293

**Published:** 2022-02-08

**Authors:** Gustavo F. Araujo, Renato Machado, Mats I. Pettersson

**Affiliations:** 1 Aeronautics Institute of Technology, São José dos Campos 12228-900, Brazil; rmachado@ita.br; 2 Blekinge Institute of Technology, 371 79 Karlskrona, Sweden; mats.pettersson@bth.se

**Keywords:** synthetic aperture radar, automatic target recognition, scattering center, classification

## Abstract

This article proposes an Automatic Target Recognition (ATR) algorithm to classify non-cooperative targets in Synthetic Aperture Radar (SAR) images. The scarcity or nonexistence of measured SAR data demands that classification algorithms rely only on synthetic data for training purposes. Based on a model represented by the set of scattering centers extracted from purely synthetic data, the proposed algorithm generates hypotheses for the set of scattering centers extracted from the target under test belonging to each class. A Goodness of Fit test is considered to verify each hypothesis, where the Likelihood Ratio Test is modified by a scattering center-weighting function common to both the model and target. Some algorithm variations are assessed for scattering center extraction and hypothesis generation and verification. The proposed solution is the first model-based classification algorithm to address the recently released Synthetic and Measured Paired Labeled Experiment (SAMPLE) dataset on a 100% synthetic training data basis. As a result, an accuracy of 91.30% in a 10-target test within a class experiment under Standard Operating Conditions (SOCs) was obtained. The algorithm was also pioneered in testing the SAMPLE dataset in Extend Operating Conditions (EOCs), assuming noise contamination and different target configurations. The proposed algorithm was shown to be robust for SNRs greater than −5 dB.

## 1. Introduction

Since the 1990s, Automatic Target Recognition (ATR) has been a very active field of study, given the diversity of its applications and the growing development of remote-sensing technologies [1]. In this context, Synthetic Aperture Radar (SAR) appears to be an outstanding tool for producing high-resolution terrain images. Such sensors stand out for three reasons: (i) radar is an active sensor that provides its own illumination, which gives it the ability to operate in the dark; (ii) clouds and rain do not prevent the passage of electromagnetic waves at common radar operating frequencies; (iii) the radar energy backscattered by different materials allows a complementary detail for target discrimination [2].

The human eye is conditioned to differentiate objects based on the reflection properties of sunlight wavelengths [3]. In turn, SAR images are formed from the backscattering of electromagnetic microwaves and their interaction with the geometry and target material. However, although rich in details, the focused image is not friendly for human eye interpretation. To successfully address this problem, it is necessary to automate the process using an intelligent computer system.

SAR ATR algorithms can be divided into three basic tasks: Pre-screening, Low-Level Classifier (LLC), and High-Level Classifier (HLC), which can also be called Detection, Discrimination, and Classification, respectively [4]. The detection task consists of extracting regions that may contain targets from an image that comprises the entire imaged area. Then, the detection output feeds the discriminator, which rejects spurious noises and clutter originating from natural and artificial formations that have characteristics different from those of the targets of interest. Finally, the classification task assigns a label that refers to the most likely class of each target candidate remaining from the discrimination task.

Over the last 30 years of research on target classification with SAR images, different approaches and algorithms have been proposed to maximize the Percentage of Correct Classification (PCC). In the literature, while some authors follow a taxonomy that separates the classification algorithms into template-based and feature-based [3], others consider template-based and feature-based to be one and suggest instead model-based and semi-model-based categories [4].

Regardless of the category of the algorithm, it is supported by features of three types: (1) Geometric features that describe the target by its area [5,6,7,8,9], contour [10,11] or shadow [11,12]; (2) Transformation features that reduce the dimensionality of the target data by representing it in another domain such as Discrete Cosine Transform (DCT) [13], Non-Negative Matrix Factorization (NMF) [14,15], Linear Discriminant Analysis (LDA) [16] and Principal Component Analysis (PCA) [17]; and (3) Scattering Centers Features which are based on the highest amplitude returns of the targets [18] and based on a statistical distance, such as Euclidean [19,20,21,22,23,24,25,26], Mahalanobis [27,28,29,30], or another statistical distance [31,32,33,34,35,36,37].

Feature-based algorithms are those with methods that run offline training supported exclusively by features extracted from the targets of interest. Among the methods employed by feature-based algorithms, we can highlight the following: Template Matching (TM) [5,6,7,11,30,37], Hidden Markov Model (HMM) [12,13,22], K-Nearest Neighbor (KNN) [27,28], Sparse Representation-based Classification (SRC) [8,29], Convolutional Neural Networks (CNN) [17,18,36,38,39,40,41,42,43,44,45,46,47,48], Support Vectors Machine (SVM) [9] and Gaussian Mixture Model (GMM) [10].

**Figure 1:**
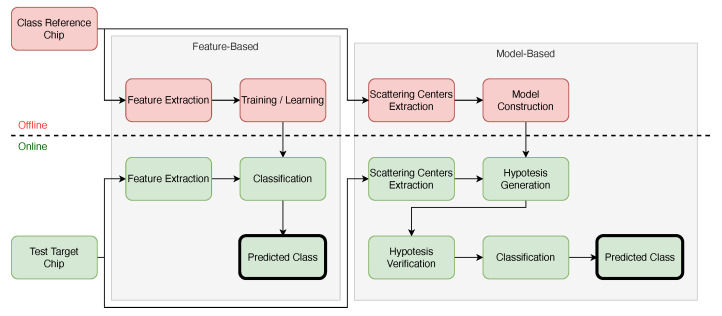
Feature and model-based classification approaches. Blocks in red and green are processed offline and online, respectively.

Model-based classifiers are distinguished from feature-based classifiers mainly by the approach adopted [19,20,21,23,24,25,26,31,32,33,34,35,49]. Model-based classifiers try to find similarities in constructed models from the image, while feature-based classifiers start from a training task to find similarities in the image. Features are extracted from potential targets, and similarities are sought in the models using hypothesis testing [4]. The tasks of a model-based classifier are performed in two steps: (i) offline construction of the class models and (ii) online prediction of the target class. During online classification, model-based methods rely on features extracted from the target candidates to generate hypotheses. Each hypothesis gives a score that assigns a target to a class from a class book. The scores are then compared, and the most likely class is identified. Figure 1 illustrates the steps of both feature-based and model-based classifications.

Some model-based algorithms, prior to feature extraction, employ the Hungarian Algorithm [23,24,29,30,31,32,33,36] to assign each scattering center of the model to a scattering center of the target. Then, features are created for both the target and the models of each class.

**Figure 2:**
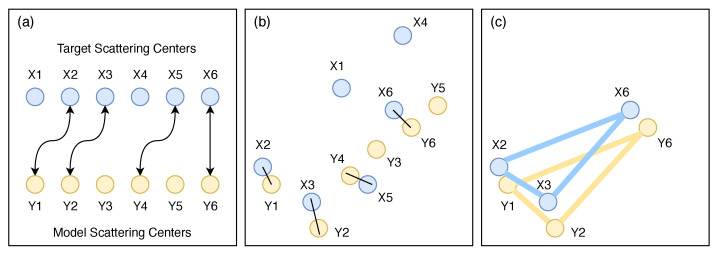
(**a**) Hungarian Algorithm scattering centers assignment, (**b**) global similarity and (**c**) local similarity.

Ding and Wen [32] used a statistical-based distance measure between pairs of scattering centers assigned by the Hungarian Algorithm to compute global similarity and triangular structures of scattering centers to identify local similarities, as shown in Figure 2.

**Figure 3:**
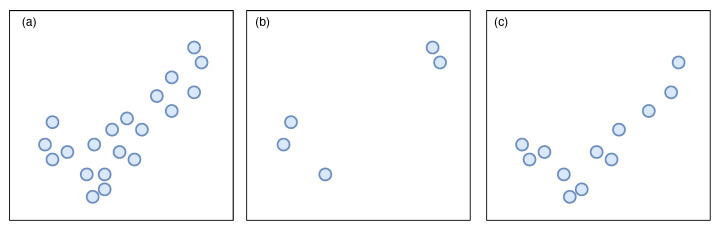
(**a**) Dominant Scattering Area of the target, (**b**) true class residues and (**c**) false class residues.

Shan et al. [49] used morphological opening and closing operations to create Dominant Scattering Areas (DSA) in the original image. Subtracting the DSA of the test image from those of different classes of models resulted in the residues representing the differences between the test image and the classes. An example is presented in Figure 3.

**Figure 4:**
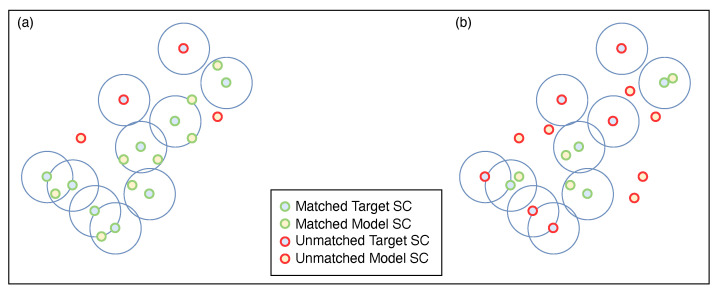
Neighbor Matching Algorithm applied to (**a**) the true class and (**b**) a false class.

Fan and Thomas [35] created masks by drawing circles centered on each scattering center of the target under test. The masks were then used by the Neighbor Matching Algorithm (NMA) to filter the scattering centers of each model class. This last approach, illustrated in Figure 4, was implemented for comparison purposes with the proposed algorithm, and the results are presented in Section 3.3.

Unlike these approaches, the proposed algorithm does not need to assign pairs of scattering centers or perform morphological or filtering operations; it is directly applicable, simple and fast, as the features are the scattering centers themselves.

This article proposes an approach to solve the problem of non-cooperative target classification in SAR images. It is taken as a premise that the targets are non-cooperative, i.e., they are not exposed frequently, so there are not enough SAR images of the targets to be used in a classification algorithm based on measured data. Therefore, the proposed approach considers only synthetic data to train the algorithm. Synthetic data, also known as simulated data, are generated through computer simulations. The most common way to produce synthetic data is by using asymptotic electromagnetic scattering prediction codes with the support of Three-Dimensional Computer-Aided Design (3D-CAD) [40].

The proposed algorithm is model-based and uses scattering centers as features. The hypotheses were verified through a Modified Goodness of Fit (MGoF) test, consisting of a weighted GoF test. The algorithm was tested by varying the following parameters—the scattering center-extraction method, the hypothesis-generation method, and the GoF test—to determine the configuration that achieved the best performance under Standard Operating Conditions (SOCs). Moreover, the performance of the algorithm was also verified under Extended Operating Conditions (EOCs), which involved images contaminated with noise and different target configurations. The works [19,20,21,23,24,25,26,31,32,33,34,35] that most resembled this one are those where the implemented algorithms aimed at the model-based classification of targets in the Moving and Stationary Target Acquisition and Recognition (MSTAR) dataset [50].

The recently released SAMPLE dataset [38], which is presented in Section 2.1, is a set of SAR images containing synthetic and measured data with very high fidelity. This dataset has great potential to become the main benchmark for SAR ATR classification algorithms, replacing or improving the MSTAR dataset, which has already been extensively explored. Therefore, there is an enormous demand for works that address the SAMPLE dataset [51]. So far, few works [38,39,40,41,42,43,44,45,46,47,48] have been carried out that focus on the SAMPLE dataset for classifying measured data based exclusively on synthetic data, and they all use a feature-based approach with machine-learning algorithms like CNN or DenseNet.

There a two main contributions from this work:

1. A simple but efficient model-based algorithm for target classification using a modified Goodness of Fit test in its decision rule that significantly reduces the dimensionality of the data because it works with a small number of scattering centers; and
2. The SAMPLE dataset was used to evaluate the proposed algorithm, the performance of which was assessed under an EOC, which considered only synthetic data for training and only measured data for testing. Various combinations of hypothesis generation and verification were evaluated.

In the remainder of this article, Section 2 details the SAMPLE dataset and the proposed SAR model-based target classification algorithm. Section 3 presents all the experiments and their results using the SAMPLE dataset. Section 4 provides insightful discussion comparing the proposed algorithm to other recent solutions addressing the SAMPLE dataset. Section 5 concludes the article.

## 2. Materials and Methods

We used a training dataset consisting exclusively of synthetic data to test the proposed algorithm assuming a non-cooperative target classification scenario. The methodology for the proposed algorithm is concerned with avoiding leaks between the training and test data sets, where the former and the latter are composed only of synthetic and measured data, respectively.

### 2.1. Materials: Measured and Synthetic SAR Data

Automatic target recognition in SAR images, when strictly based on synthetic data, must meet certain requirements so that experiments can achieve satisfactory results. An evident and natural requirement is the similarity of synthetic data when compared to the measured data. The more similar the synthetic and measured data, the more similar the features used by the classification algorithms.

Electromagnetic Computing (EMC) systems allow the simulation of a scenario where a 3D-CAD, representative of a target, interacts with radar signals. By properly setting the radar’s parameters and trajectory, the backscatter signal can be recorded, and then a simulated image of the target can be obtained using SAR processing. In addition, it is essential that appropriate EMC algorithms be used and that the physical characteristics of the simulated target be as close as possible to those of the real target [26].

Thanks to the Air Force Research Laboratory and Wright State University, a valuable tool has recently been made available to the scientific community: the Synthetic and Measured Paired and Labeled Experiment (SAMPLE) dataset [38], which consists of an upgraded MSTAR dataset [50], previously made available in 1995. The great difference in the SAMPLE dataset lies in the fidelity of the physical characteristics of the targets introduced in the simulation. Thorough work was carried out in the SAMPLE dataset to determine the geometric shapes, roughness, and dielectric characteristics of each section of the targets.

**Figure 5:**
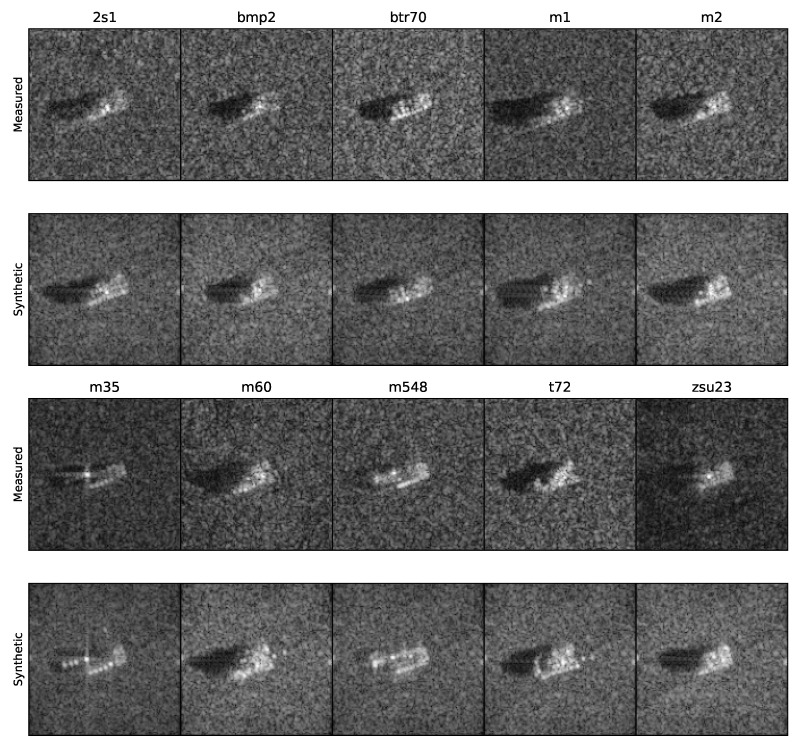
Example of one image of each vehicle in the SAMPLE dataset. Measured MSTAR images are on the top row, and the corresponding synthetic images are on the bottom row. The order of the vehicles from left to right is the same as presented in Table 1. We see that details such as shadows, orientation, and relative return magnitudes are in good agreement [38,44].

The SAMPLE dataset contains 10 target classes that had been preprocessed, resulting in complex SAR image chips of 128 × 128 pixels. For each measured data chip obtained by the X-band sensor, a synthetic data chip was available (respecting the same depression and azimuth angles). In one-degree increments, the dataset chips discontinuously ranged from 10 to 80 degrees in azimuth and from 14 to 17 degrees in depression angle. The 10 classes from the SAMPLE dataset are listed in Table 1, and examples of both measured and synthetic data are shown in Figure 5.

Considered state of the art for simulating SAR images, the SAMPLE dataset was used in this work. As presented in Section 2.2.2, the synthetic data were used as inputs to generate a model based on scattering centers to feed the classification algorithms. The measured data were considered for classification and to evaluate the proposed algorithm.

### 2.2. Methods

One of the greatest difficulties in working with target recognition in SAR images lies in the nature of the sensor. Two images of the same target obtained by the same optical sensor but with small variations in aspect angles still have a significant similarity. However, when it comes to a SAR sensor, small variations in aspect angles substantially change the resulting image [19].

Many studies on SAR ATR are being developed focusing on machine learning. In training a class, such an approach uses a set of measured data chips with different aspect angles representative of the same class. Suspecting that the great distinction of SAR images of the same target as a function of aspect angles may cause an unwanted bias, we decided not to use a machine-learning-based approach. Instead, we proposed a model-based algorithm.

**Figure 6:**
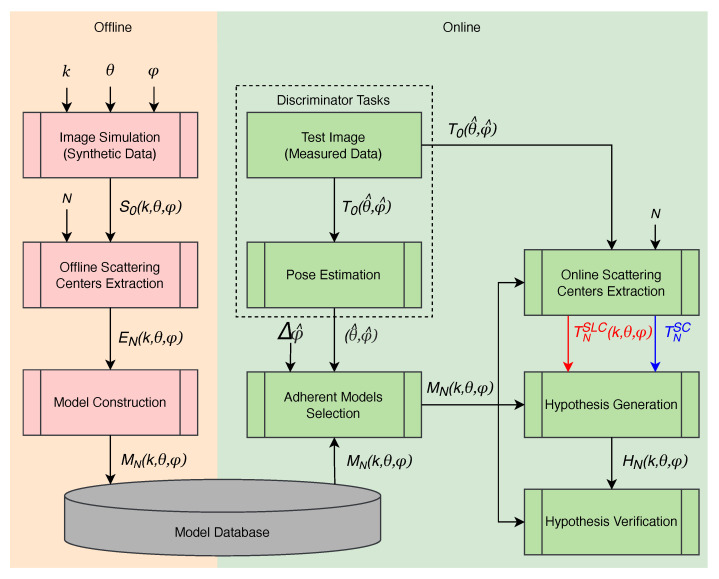
General design of the proposed classification algorithm.

The classification algorithm proposed in this article (detailed in Figure 6) does not use a set of measured data chips to train a specific class. Basically, the algorithm training consists of creating a model, strictly based on synthetic data, for each combination of class (*k*) and aspect angles (θ,ϕ) and then storing them in a database. The classification task is performed online to verify the generated hypotheses based on the most adherent models in the database.

#### 2.2.1. Image Simulation

**Figure 7:**
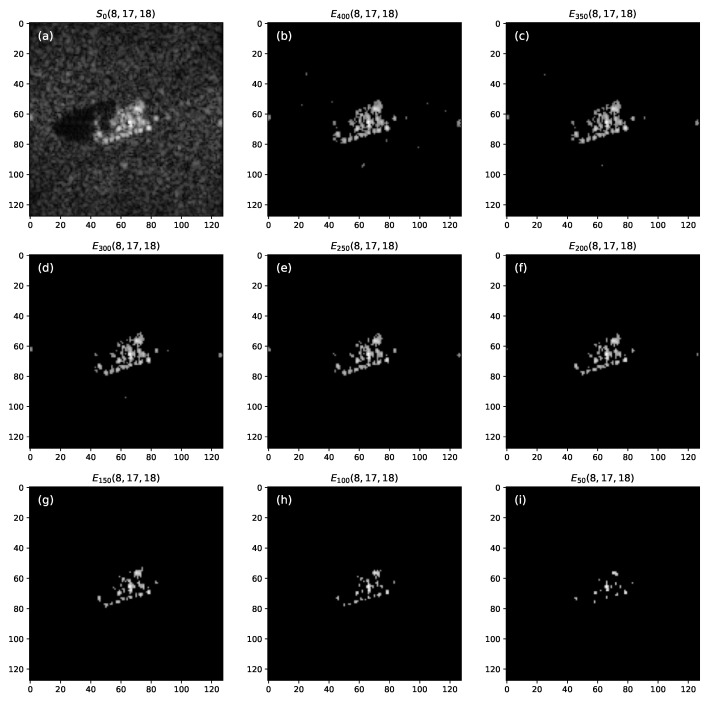
(**a**) Synthetic data chip of a T-72 tank—$S_{0}\left(8,17,18\right)$. Reconstructed image with (**b**) 400, (**c**) 350, (**d**) 300, (**e**) 250, (**f**) 200, (**g**) 150, (**h**) 100, and (**i**) 50 scattering centers.

The offline tasks start with simulations of the SAR images for each class (*k*) of a set (*K*). The completeness of the model database depends on the range (Θ,Φ) of aspect angles (θ,ϕ) and the spacing between adjacent chips (Δθ,Δϕ). The higher the depression angle (θ) span, the greater the probability that chips extracted at the edges of the image range axis will be correctly classified. Furthermore, the larger the azimuth angle (ϕ) span, the greater the range of target poses the algorithm can correctly classify. The spacing between adjacent chips (Δθ,Δϕ) plays an important role in model accuracy, considering that even small angular variations greatly distort a SAR image. At the end of this task, there is a synthetic data chip $S_{0}\left(k,\theta ,\phi \right)$ for each combination of class, depression angle, and azimuth angle. Since each class presents a slightly different value in its azimuth angle (ϕ) decimals, this angle is rounded to the nearest integer to become a common label reference. Figure 7a shows an example of a T-72 class (k=8) synthetic-target data at $17^{\circ }$ depression angle and $17.77^{\circ }$ azimuth angle $S_{0}\left(8,17,18\right)$.

#### 2.2.2. Offline Extraction of Scattering Centers

The proposed algorithm performs target classification, the last stage of an automatic target-recognition activity. Therefore, it is assumed that the targets to be classified already went through the detection and discrimination stages. Consequently, the target candidates are already centered on the chips to be tested. This task aims to reduce the dimension of the data used for classification, providing greater processing speed and more accurate results. The idea here is to use a sparse image representation based on the parts of the target that are sufficient for its characterization.

According to Potter and Moses [52], the high-frequency scattering response of a distributed object is well approximated as a sum of responses from individual scatterers, or scattering centers, which are good candidates to be used in automatic target recognition. Their research resulted in an established model for the backscattered electric field in the frequency-aspect domain

(1) $$E\left(f,\theta \right)=\sum_{n=1}^{N}A_{n}{\left(\frac{jf}{f_{c}}\right)}^{\alpha_{n}}\exp\left\{\beta_{n}\theta \right\}\exp\left\{\frac{-j4\pi f}{c}\left(x_{n}\cos\theta +y_{n}\sin\theta \right)\right\},$$

where $f_{c}$ is the center frequency, and the parameters of the *n*th scattering center are the complex magnitude and phase ($A_{n}$), slant plane location ($x_{n},y_{n}$), geometry/frequency dependence ($\alpha_{n}$) and angular dependence ($\beta_{n}$).

Some algorithms can be used to extract scattering centers in the image domain [53]. The CLEAN algorithm, although originally developed for applications in radio astronomy [54], is one of the most used for extracting scattering centers on SAR images [8,23,31,53,55].

The CLEAN algorithm employs a filter derived from the Point Spread Function (PSF), which is given by

(2) $$psf\left(x,y\right)=\left(e^{j\frac{4\pi f_{c}}{c}\left(x+\theta_{c}y\right)}\frac{4f_{c}B\Omega }{c^{2}}\right)\mathrm{sinc}\left(\frac{2B}{c}x\right)\mathrm{sinc}\left(\frac{2f_{c}\Omega }{c}y\right),$$

where (x,y) denotes the position of the scattering center; $f_{c}$ is the center frequency; $\theta_{c}$ is the center azimuth; *B* is the frequency bandwidth of the radar; and Ω is the azimuth aperture.

To extract the scattering centers properly, the filter used in the CLEAN algorithm had to incorporate the same smoothing window w(x,y) used during image formation, resulting in

(3) h(x,y)=psf(x,y)w(x,y).

**Figure 8:**
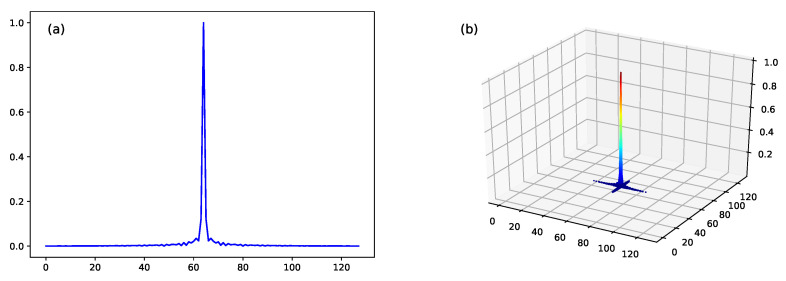
Filter used by CLEAN algorithm with SAMPLE dataset. (**a**) 2D plot; (**b**) 3D plot.

This work used a −35 dB Taylor Window [56], the same one employed by the SAMPLE dataset [38]. The PSF is set with the radar parameters specified in Table 2. The filter used by the CLEAN algorithm is illustrated in Figure 8.

For the scattering center extraction procedure, the CLEAN algorithm searches for the highest amplitude pixel in the image, recording both the amplitude ($A_{n}$) and its coordinates ($x_{n},y_{n}$). Then the filter h(x,y) shifts to the center of the pixel location and is multiplied by $A_{n}$, resulting in $A_{n}h\left(x-x_{n},y-y_{n}\right)$, which is subtracted from the image. Those steps are repeated with the residual image until all *N* scattering centers have been extracted. The entire process can be written as

(4) $$S_{n}\left(k,\theta ,\phi \right)=\left\{S_{n-1}\left(k,\theta ,\phi \right)-A_{n}h\left(x-x_{n},y-y_{n}\right)|n=1,2,\ldots ,N\right\},$$

where $S_{n}\left(k,\theta ,\phi \right)$ is the resulting image after the *n*th scattering center has been extracted from the $S_{n-1}\left(k,\theta ,\phi \right)$ image.

Typically, the total energy of the residual image drops sharply after a few interactions. At the end of the extraction process, the scattering centers are stored in an N×3 matrix, $E_{N}\left(k,\theta ,\phi \right)=\left\{A_{n},x_{n},y_{n}|n=1,2,\ldots ,N\right\}$, the sparsity of which provides a great reduction in data storage compared to the original complex image. Data compression rates greater than 60:1 can be easily achieved [57]. As an example, Figure 7b–i presents reconstructed images with different amounts of scattering centers extracted from the simulated image.

#### 2.2.3. Model Construction

The proposed algorithm uses a score obtained from the GoF test, which has the scattering centers amplitude as inputs. The ratio between each scattering center and the sum of all scattering centers amplitudes is registered for the models

(5) $$M_{N}\left(k,\theta ,\phi \right)=\left\{\frac{A_{n}}{\sum_{i=1}^{N}A_{i}},x_{n},y_{n}|n=1,2,\ldots ,N\right\}.$$

All models are stored in a database, which is accessed later during online classification (Section 2.2.5) to extract the proper scattering centers from the measured testing data to generate a classification hypothesis.

**Figure 9:**
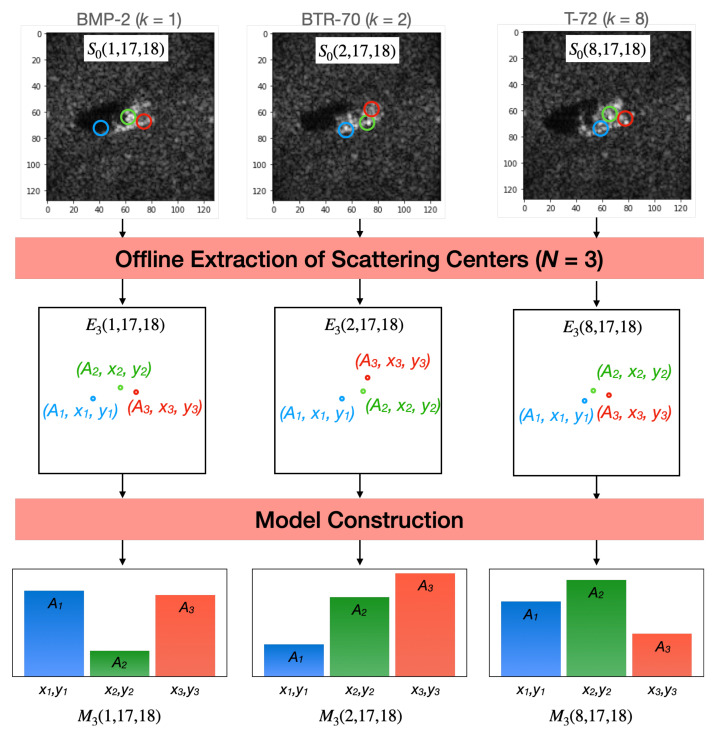
Illustrative example of the construction of three different class models (BMP-2, BTR-70 and T-72) at the same aspect angles ($\theta =17^{\circ }$ and $\phi =18^{\circ }$) with three scattering centers each.

Figure 9 illustrates the construction of models for classes BMP-2, BTR-70, and T-72 considering the depression and azimuth angles of $17^{\circ }$ and $18^{\circ }$ respectively. It exemplifies a hypothetical case where only three scattering centers are extracted from each simulated image.

#### 2.2.4. Discriminator Tasks

Online classification starts after receiving a test chip $T_{0}\left(\hat{\theta },\hat{\phi }\right)$ from the discriminator stage. For the success of the classification, image registration must be ensured by the discriminator since the precise location of each part of the target has a large impact on the Percentage of Correct Classification (PCC). In addition, the discriminator needs to provide an accurate estimate of the azimuth angle ($\hat{\phi }$) of the longitudinal axis of the candidate target. In this article, it is assumed that the depression angle ($\hat{\theta }$) can be calculated since the distance and relative height between the radar platform and the target are known.

In the SAMPLE dataset, the targets are perfectly centered on chips and labeled with the depression (θ) and azimuth (ϕ) angles in which they were simulated, thus meeting the requirements of the discriminator stage.

#### 2.2.5. Selection of Adherent Models

Taking the estimated aspect angles ($\hat{\theta },\hat{\phi }$) as inputs, this task aims to find the most relevant models $M_{N}\left(k,\theta ,\phi \right)$ in the database to be used in the hypothesis generation task. Thus, each selected model will lead to a hypothesis to be tested further and confronted with the others.

The $\Delta \hat{\phi }$ parameter defines the error tolerance in estimating the azimuth angle of the target. Therefore, all models that have the same depression (θ) and azimuth angle (ϕ) within $\hat{\phi }\pm \Delta \hat{\phi }$ will be selected as adherent models $D_{N}\left(k,\theta ,\phi \right)$.

While observing the images from the SAMPLE dataset (Figure 5), we saw a great similarity between the front and rear faces of each target. The difficulty in distinguishing them entails a 180-degree ambiguity. Thus, models with azimuth angles (ϕ) within $180+\hat{\phi }\pm \Delta \hat{\phi }$ were also selected. In the SAMPLE dataset, this ambiguity did not apply, since the azimuth angles of its chips were limited to a range from 10 to 80 degrees.

#### 2.2.6. Online Extraction of Scattering Centers

Two different means of hypothesis generation were implemented, as presented in Section 2.2.7 and analyzed in Section 3. The difference between these approaches is in how the chip data were handled. Each approach used a different source to extract the scattering centers to deliver distinct inputs to the hypothesis-generation task. Both approaches— Single Look Complex (SLC)-based and Scattering Center (SC)-based—provided the Hypothesis Generation with the location of the scattering centers, which were in the same coordinates of the model.

The SC-based approach performed scattering centers extraction from the measured data in the same way as it was done offline for the synthetic data, as described in Section 2.2.2. No matter how many models were selected in the Selection of Adherent Models task (Section 2.2.5), only a single set of *N* scattering centers $T_{N}^{SC}$ were extracted and used in the Hypothesis Generation task.

Conversely, the SLC-based approach extracted a set of *N* scattering centers for each adherent model $D_{N}\left(k,\theta ,\phi \right)$. These extractions were carried out in a guided manner. The CLEAN method still extracted the highest amplitude scattering centers. However, it only saved the scattering centers located in the same coordinate pair (x,y) of the model. For each adherent model $D_{N}\left(k,\theta ,\phi \right)$ selected by the Selection of Adherent Models task (Section 2.2.5), a set of scattering centers $T_{N}^{SLC}\left(k,\theta ,\phi \right)$ was created to be used later for hypothesis generation.

#### 2.2.7. Hypothesis Generation

As previously stated, different approaches to extracting scattering centers produce different sources as inputs for generating hypotheses.

In the SLC-based approach, each set $T_{N}^{SLC}\left(k,\theta ,\phi \right)$ resulting from the Online Extraction of Scattering Centers task generated a hypothesis $H_{N}\left(k,\theta ,\phi \right)$ by computing the amplitudes ratio in the same way as in Section 2.2.3—Model Construction, as given by Equation (5).

**Figure 10:**
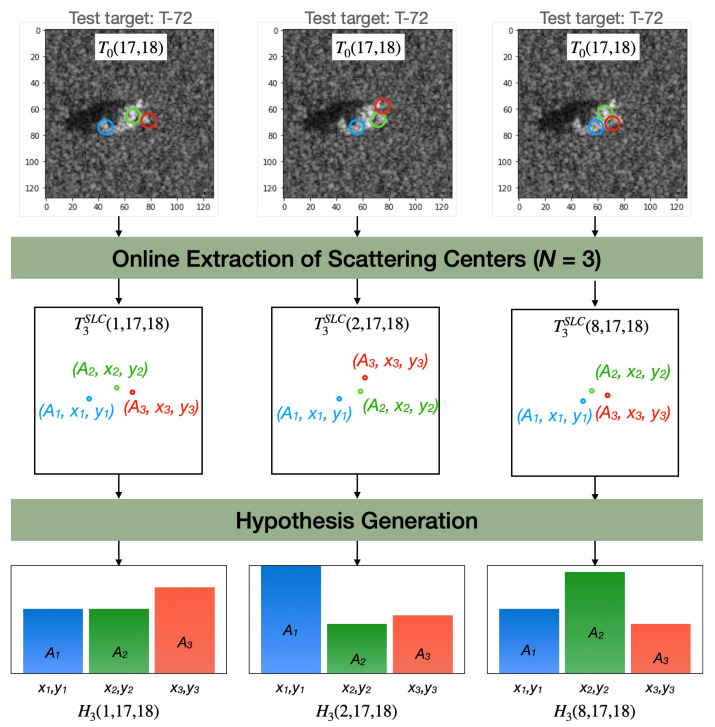
Illustrated example of three hypotheses generation by the SLC-based approach. Each hypothesis (BMP-2, BTR-70 and T-72 classes) was generated by extracting three scattering centers from the T-72 test target accordingly to the respective class model.

Figure 10 illustrates an example of the SLC-based approach, where a test target $T_{0}\left(17,18\right)$ has its scattering centers $T_{3}^{SLC}\left(k=\left[1,2,8\right],17,18\right)$ extracted according to three different models, resulting in three hypotheses $H_{3}\left(k=\left[1,2,8\right],17,18\right)$.

For the SC approach, the input source was a single set $T_{N}^{SC}$ containing the *N* highest scattering centers in the test image. Thus, a complete match between their locations and those in the selected models was not likely. Therefore, if a $\left(x_{n},y_{n}\right)$ pair of the model $D_{N}\left(k,\theta ,\phi \right)$ existed in the extracted scattering centers $T_{N}^{SC}$, the amplitude of the test image scattering center was assigned to the hypothesis under construction $T_{N}^{SC}\left(k,\theta ,\phi \right)$. Otherwise, zero was assigned as the amplitude. In the end, as considered in the previous approach, the Equation (5) was applied to calculate the ratio between the amplitudes. Then hypothesis $H_{N}\left(k,\theta ,\phi \right)$ was generated.

Although the SC-based approach handled a smaller amount of information, we will see in Section 3.1.2 that it resulted in a considerable improvement in the percentage of correct classification performance.

**Figure 11:**
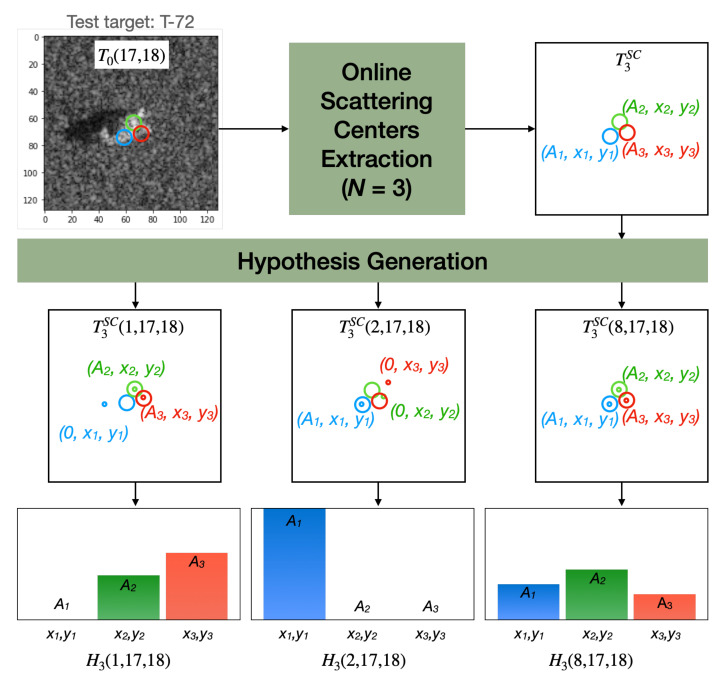
Illustrated example of the three hypotheses generated by SC-based approach. Each hypothesis (BMP-2, BTR-70, and T-72 classes) was generated by extracting three scattering centers from the T-72 test target accordingly to the respective class model.

Figure 11 illustrates an example of the SC-based approach where a test target $T_{0}\left(17,18\right)$ has its scattering centers with the highest amplitudes extracted $T_{3}^{SC}$. It also resulted in three hypotheses $H_{3}\left(k=\left[1,2,8\right],17,18\right)$ with amplitudes defined by the location of the scattering centers of the models.

#### 2.2.8. Hypothesis Verification

Once each hypothesis referring to a selected model has been generated, the final task is to predict the class of the target under test. For this purpose, the verification of the hypotheses is carried out through a Goodness of Fit (GoF) test.

**Figure 12:**
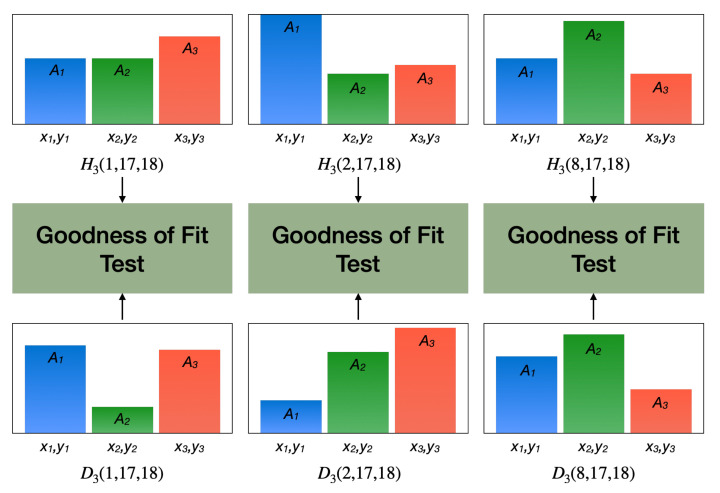
Illustrated example of hypothesis verification where the test target (T-72 class) is confronted against three hypotheses (BMP-2, BTR-70, and T-72 classes).

Three testing algorithms were initially implemented: Mean Square Error (MSE), Pearson’s Chi-Square (PXS), and the Likelihood Ratio Test (LRT). Considering that each pair formed by a model $D_{N}\left(k,\theta ,\phi \right)$ and a hypothesis $H_{N}\left(k,\theta ,\phi \right)$ had its own scattering centers located at the same coordinates (x,y), a Goodness of Fit test was run for each, as depicted in the hypothetical example of Figure 12.

The MSE test score of a model and hypothesis pair is given by

(6) $$P_{N}^{MSE}\left(k,\theta ,\phi \right)=\sum_{n=1}^{N}{\left(A_{H_{n}}-A_{D_{n}}\right)}^{2},$$

where $A_{D_{n}}$ and $A_{H_{n}}$ are the expected and real amplitudes of the *n*th scattering centers.

Pearson’s Chi-Square Test is a normalization of the MSE Test, where the score is calculated by

(7) $$P_{N}^{PXS}\left(k,\theta ,\phi \right)=\sum_{n=1}^{N}\frac{{\left(A_{H_{n}}-A_{D_{n}}\right)}^{2}}{A_{D_{n}}},$$

while the Likelihood Ratio Test (LRT) score can be computed as

(8) $$P_{N}^{LRT}\left(k,\theta ,\phi \right)=2\times \sum_{n=1}^{N}\left(A_{H_{n}}+\epsilon \right)\ln\left(\frac{A_{H_{n}}+\epsilon }{A_{D_{n}}}\right).$$

A constant ϵ was added to $A_{H_{n}}$ to prevent the computation of an undefined logarithm when the hypothesis measured amplitude was equal to zero. The value assigned to the constant also acted as a weight for the influence of null scattering centers on the score. The constant was empirically determined as

(9) $$\epsilon =\left\{\begin{array}{cc} 0, & \mathrm{if}\;A_{H_{n}}\neq 0\\ 10^{-10}, & \mathrm{otherwise}. \end{array}\right.$$

The fourth GoF test was proposed as a variation of the LRT, considering a particularity of the SC-based approach. The set of scattering centers extracted from the test image was unique ($T_{N}^{SC}$). It was expected that the majority of the scattering centers in this set would also be found in the true-class model. Therefore, a weight inversely proportional to the number of scattering centers found in the same (x,y) coordinate pair of both the model and hypothesis was incorporated into the GoF test. As will be shown in Section 3, since the LRT achieved the best results of all GoF tests, its weight was used in the Modified Likelihood Ratio Test (MLRT) as

(10) $$P_{N}^{MLRT}\left(k,\theta ,\phi \right)=\frac{2}{N_{D\cap H}}\sum_{n=1}^{N}\left(A_{H_{n}}+\epsilon \right)\ln\left(\frac{A_{H_{n}}+\epsilon }{A_{D_{n}}}\right),$$

where $N_{D\cap H}$ is the amount of co-located scattering centers found in both the model and hypothesis. Figure 11 presents an illustrative example, where $N_{D\cap H}$ is equal to 2, 1, and 3 for $P_{3}^{MLRT}\left(1,17,18\right)$, $P_{3}^{MLRT}\left(2,17,18\right)$ and $P_{3}^{MLRT}\left(8,17,18\right)$, respectively.

The scores resulting from the GoF tests, performed for each model and hypothesis pair, are compared. The lower the score of a given test, the greater the similarity between the model and the hypothesis. In this work, where only Within Class (WIC) classification was considered, the hypothesis verification that resulted in the lowest score determined the target’s predicted class.

(11) $$\hat{k}=\arg\min_{k_{i}}P_{N}\left(k_{i},\theta ,\phi \right).$$

However, in a test where confusers are present, a threshold ($P_{N}^{tshd}$) should be adjusted such that

(12) $$\hat{k}=\left\{\begin{array}{cc} k_{i}, & \mathrm{if}\;P_{N}\left(k_{j},\theta ,\phi \right)-P_{N}\left(k_{i},\theta ,\phi \right)>P_{N}^{tshd}|\forall k_{j}\in K,\theta \in \Theta ,\phi \in \Phi ,i\neq j\\ \mathrm{confuser}, & \mathrm{otherwise}. \end{array}\right.$$

## 3. Results

As stated in Section 2.1, the Standard Operating Condition (SOC) experiments were designed based on the SAMPLE dataset to use the maximum amount of chips available from each class. However, although the dataset contained image chips for the 10 classes, for $\theta =[14^{\circ },17^{\circ }]$ ($\Delta \theta =1^{\circ }$) and for $\phi =[10^{\circ },80^{\circ }]$ ($\Delta \phi =1^{\circ }$), this range was discontinuous. In other words, there were no images with certain aspect angles for some classes. The aspect angles (θ,ϕ) of all 10 classes available in the data set were identified. The 23 pairs of aspect angles used in the experiments are listed in Table 3.

### 3.1. Standard Operating Condition

A total of 230 chips were taken to generate the selected models according to Table 3. From these chips, models with 50, 100, 150, 200, 250, 300, 350, and 400 scattering centers were built by applying the CLEAN scattering center extraction algorithm. GoF test experiments were performed to evaluate the classification methods.

#### 3.1.1. Goodness of Fit Test Experiments

In this analysis, an experiment was performed for each GoF test: MSE, PXS, and LRT in 10-class within class classification tests by using the SLC-based approach as the source for hypothesis generation.

The experiment considered 24 configurations evaluated through the GoF tests. For each combination of a test and a different number of scattering centers in the model, all 230 chips underwent classification.

**Figure 13:**
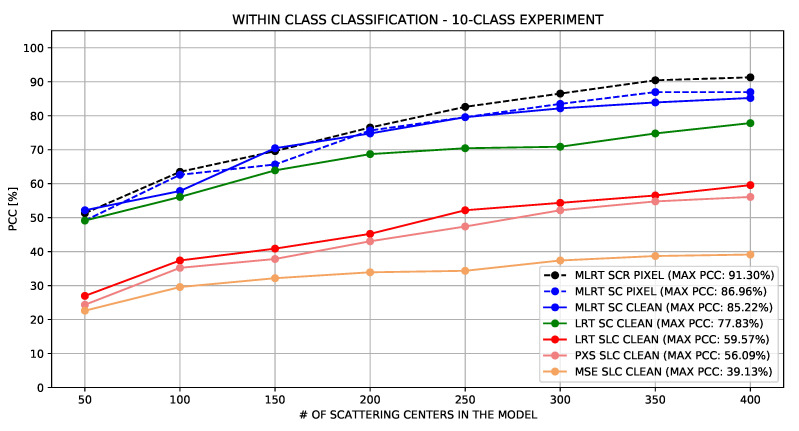
Standard Operating Condition—Classification resulting from different implementations.

The first three columns of Table 4 summarize the maximum PCC obtained by each GoF test. As can be seen, regardless of the number of scattering centers within the model, the LRT stood out in the results, obtaining the highest PCC. The results were also shown by the three lowest curves in Figure 13.

#### 3.1.2. Hypothesis Generation Source Experiments

In recognition of the superiority of its results, the LRT was selected to be used as the reference to compare sets obtained through different hypothesis generation approaches, i.e., SLC- and SC-based (Section 2.2.7). The same 24 settings from Section 3.1.1 were used to assess the performance of these two different approaches.

Figure 13 also presents the LRT SC-based approach results, which, as can be seen, (green curve) outperformed the SLC-based approach (red curve) in all configurations.

The fourth column of Table 4 shows that the results obtained by the LRT SC-based approach were superior to the SLC-based approach, providing a PCC that was up to 23% higher.

#### 3.1.3. Modified Likelihood Ratio Test Experiment

In this experiment, the performances obtained from the proposed GoF and MLRT tests were assessed. As can be seen in the solid blue line of Figure 13 and the fifth column of Table 4, the PCC achieved by the MLRT test presented gains of up to 11% over the LRT.

#### 3.1.4. Modified CLEAN Extraction Method Experiment

Since the CLEAN algorithm performs a large number of operations in the extraction of each scattering center (Equation (4)), its implementation may be unfeasible in real-time applications where a quick system response is expected. For example, considering a 128×128 pixel chip, the original PSF would have to perform 16,384 operations for each scattering center extraction. An alternative for speeding up the execution of the CLEAN algorithm was proposed: limiting the filter kernel (PSF) (Figure 8) to a single pixel, i.e., the central one, by replacing the PSF for the impulse function δ(x,y). Thus, only one operation was performed in the extraction of each scattering center. The proposed method was named PIXEL.

An experiment was carried out to verify how the PIXEL extraction method affected the PCC. The dashed blue line in Figure 13 illustrates the results obtained when the PIXEL method was used as the filter kernel in the extraction of scattering centers. The sixth column of the Table 4 shows that there were no significant changes in results compared to the original CLEAN method. On the contrary, a small performance improvement was acquired with the simplified kernel.

#### 3.1.5. Scattering Centers Reduction Based Approach Experiment

The SC-based approach achieved a good performance in hypotheses generation by increasing accuracy by up to 16%. A modification of this approach, called Scattering Centers Reduction (SCR), was proposed as a variant. For the models with *N* scattering centers, the SC-based approach assigned zero to the scattering centers amplitudes that were not ranked within the *N* highest ones. The SCR-based approach was motivated by the possibility of assigning zero to an even greater number of scattering centers on the test chip. Thus, the SCR-based experiment was carried out by varying the number of non-zero amplitude scattering centers from 1 to *N* on the measured data chip.

**Figure 14:**
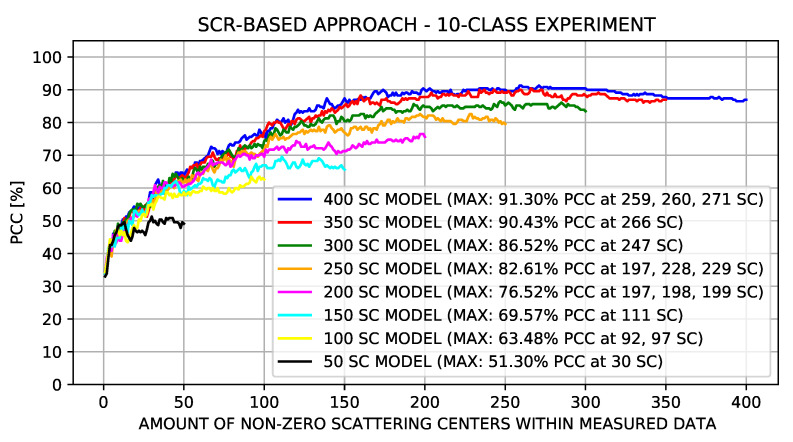
SCR-based approach experiments considering the number of non-zero scattering centers in the measured data.

Figure 14 shows the results of the SCR-base approach experiments in conjunction with the MLRT. The number of scattering centers of the models (*N*) was 50, 100, 150, 200, 250, 300, 350, and 400, while the number of non-zero scattering centers of the measured data chips ranged from 1 to *N*.

It can be seen in Figure 14 that for the results of all sets greater than 250, the maximum PCC points lay between 247 and 271 scattering centers. From this range, when the number of scattering centers increased, the PCC decreased. Thus, we can infer that the average number of scattering centers representative of the target is within this range.

The last column of Table 4 and the dashed black line of Figure 13 show the results obtained from the SCR-based approach.

Table 5 presents the confusion matrix resulting from the experiment carried out according to model size in which the highest PCC were verified, i.e., 400 extracted scattering centers. The results were obtained using the PIXEL extraction method, SCR-based approach, and MLRT. An overall PCC of 91.30% was acquired.

### 3.2. Extended Operating Condition

When dealing with SOCs, synthetic data presented maximum similarity to the measured data, and image acquisition was considered ideal as it lacked real-system degradation. In the real world, targets found in the images can present relevant variations in their geometry and in the materials that comprise them. The SAR imaging system is also subject to a wide range of factors that can affect the quality of the images, whether they are inherent to the system or arise from the natural conditions of the environment. Then, Extended Operating Conditions (EOCs) were considered in two experiments to verify the proposed algorithm under conditions where either there were structural differences between the measured and the synthetic data or the image was contaminated by noise.

#### 3.2.1. Noise Corruption Experiments

Noise Corruption Experiments were performed to evaluate the proposed algorithms under different Signal-to-Noise Ratios (SNRs). The average noise power is given by the difference between the average power of the measured data ($E[T_{0}{\left(k,\theta ,\phi \right)}^{2}]$) in dB and the desired SNR. As the average power of noise is equal to the noise variance, $E\left[N_{SNR}^{2}\right]=\mu^{2}+\sigma^{2}$, and the average noise is equal to zero, the added Gaussian noise can be approximated by

(13) $$N_{SNR}\left(k,\theta ,\phi \right)∼N\left(0,10^{\frac{10\log\left(E[T_{0}{\left(k,\theta ,\phi \right)}^{2}]\right)-SNR}{10}}\right).$$

**Figure 15:**
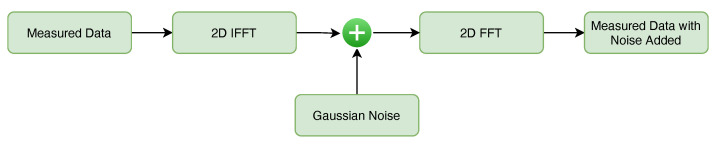
Addition of noise do the measured data.

Before the Gaussian noise is added, the measured data is transformed into the frequency and aspect domain to reflect the raw data. Afterwards, the noise is added to simulate the phenomenon that is supposed to occur during data acquisition. Finally, the data are transformed back into the range and cross-range domains. The noise addition procedure is illustrated in Figure 15.

**Figure 16:**
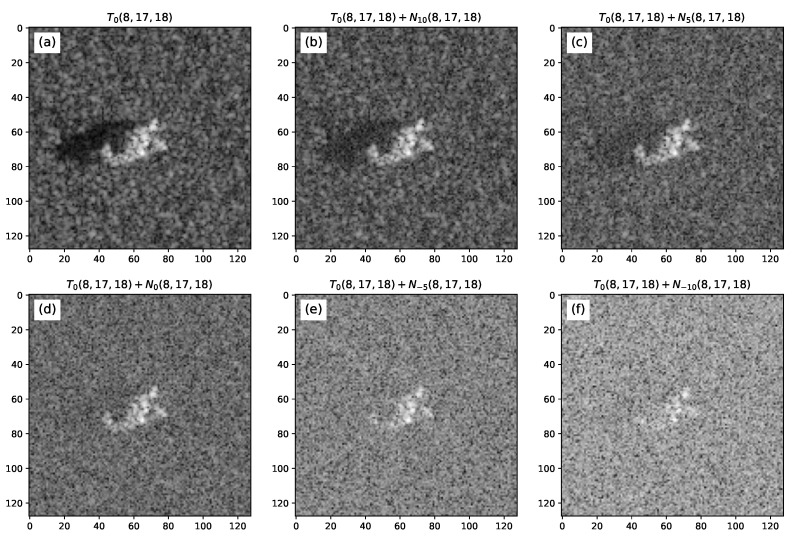
(**a**) Measured data chip of a T-72 tank—$T_{0}\left(8,17,18\right)$. Gaussian noise added relative to either (**b**) 10 dB, (**c**) 5 dB, (**d**) 0 dB, (**e**) −5 dB and (**f**) −10 dB of SNR.

The corrupted images were simulated for the SNR = 10, 5, 0, −5 and −10 dB. Figure 16 illustrates an example of the resulting noise-contaminated images relative to each desired SNR.

Table 6 details the accuracy obtained from each set of models for SNRs.

**Figure 17:**
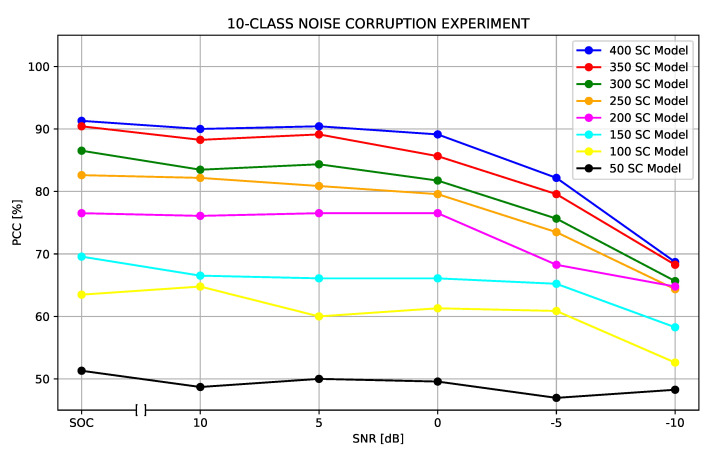
Classification results considering the addition of white Gaussian noise. The results were obtained for SNR = +10, +5, 0, −5 and −10 dB.

Figure 17 illustrates the results presented in Table 6. Note that the results are still reliable when the noise had an SNR greater than −5 dB. However, when the SNR reached −5 dB the noise started to compromise the classification results, decreasing the PCC significantly.

#### 3.2.2. Configuration Variant Experiment

In the SAMPLE dataset, all images of the same class were obtained from a single-class instance. To carry out the Configuration Variant Experiment, measured data from the MSTAR dataset were used since they had different instances of targets that had classes found in the SAMPLE dataset.

In the MSTAR dataset, the BMP-2 and T-72 classes had two vehicles with different serial numbers. While in the SAMPLE dataset, the models of these targets were built based on vehicles with the serial number 9563 (BMP-2) and 812 (T-72), in the MSTAR dataset, there were also images of the BMP-2 vehicles with serial numbers 9566 and C21, and images of T-72s with serial numbers 132 and S7. A total of 69 chips (34 BMP-2 and 35 T-72) were found in the MSTAR dataset that could be used in a within-class classification experiment, considering the two classes.

According to [50] the main characteristics that differentiate these targets of the same classes are related to configuration, articulation, and damage. For example, the different versions of the T-72 stand out for the presence or absence of fuel drums and side skirts.

**Figure 18:**
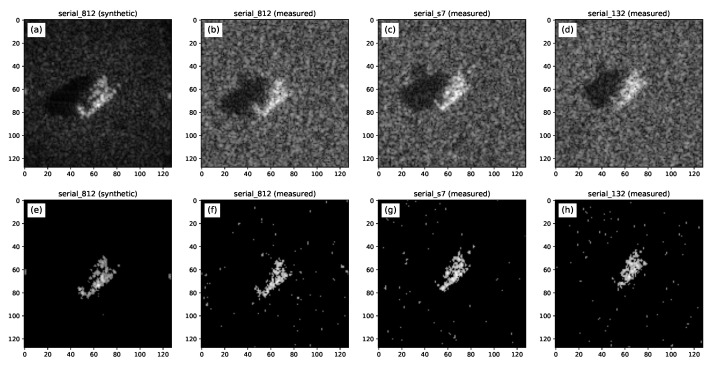
(**a**) Synthetic data chip of a T-72 tank (SN 812). Measured data of a T-72 tank of serial number: (**b**) 812, (**c**) S7 and (**d**) 132. Image reconstructed with 400 Scattering Centers extracted from (**e**) the synthetic data of the T-72 tank of serial number 812 and the measured data of the T-72 tanks of serial numbers (**f**) 812, (**g**) S7 and (**h**) 132.

Figure 18 depicts images of targets from the same class (T-72), but with different serial numbers. Note that, in this example, the synthetic data from the SAMPLE dataset based on the serial number 812 of the T-72 target (Figure 18a) was used to build the model (Figure 18e) and the MSTAR dataset measured data referring to the T-72 targets of serial number S7 and 132 (Figure 18c,d) were used in the classification experiment (Figure 18g,h).

The results were verified for both Standard and Extended Operating Conditions through Goodness of Fit tests, scattering centers extraction methods, and hypothesis generation approaches.

**Figure 19:**
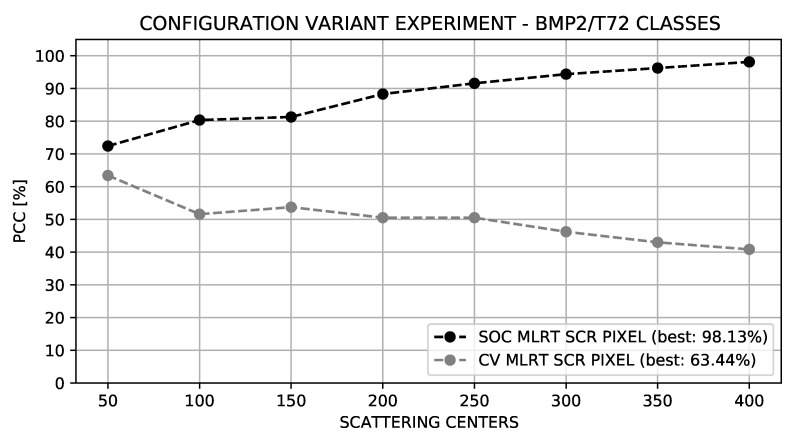
Comparison between the Configuration Variant classification and Standard Operating Condition.

Figure 19 details the comparative results between the SOC and EOC (Configuration Variance) experiments. Due to the relevant variations between targets of different serial numbers, a reduction in the PCC in the Configuration Variance Experiment was expected. Note that in addition to the expected degradation of the overall results, the performance of the Configuration Variance Experiment increases with the reduction in the number of scattering centers of the model, while the performance in the SOC enhances with the increase in the number of scattering centers of the model.

The opposite performance between SOC and EOC regarding the number of scattering centers was due to the differences between the configurations. Two targets of the same class, differently configured, presented great similarities between their basic cores; that is, a limited number of scattering centers. As the number of scattering centers is increased, fine details were incorporated into the model. Therefore, as these fine details were not common to the different configurations, there was no match between the scattering centers; consequently, the score was affected.

### 3.3. Neighbor-Matching Algorithm

As introduced in Section 1, the NMA considers the proximity of the scattering centers of the targets to those of the model, as shown in Figure 4.

NMA creates a binary region (mask) by drawing circles of radius *R* centered on each scattering center of the target image ($I_{T}$). When a model scattering center is in the constructed binary region, it is selected; otherwise, it is discarded. A model image ($I_{M}$) is reconstructed for each model with the selected scattering centers for the target under test.

The number of circumscribed target scattering centers ($M_{S}$) was counted by centering the circumferences on each scattering center of the model. Finally the normalized similarity was computed as follows:

(14) $$f_{s}\left(I_{T},I_{M}\right)=Cor\left(I_{T},I_{M}\right)\times \frac{M_{S}}{M},$$

where *M* is the total amount of scattering centers, and $Cor(I_{T},I_{M})$ is the correlation coefficient between the target image and the reconstructed model image.

In their original work, Fan and Thomas [35] addressed the MSTAR dataset that obtained a PCC of 99.04% in a 10-class experiment. However, their models were built with measured data rather than synthetic data. The radius of the circles assumed values of 0.3, 0.4, and 0.5 m. The number of scattering centers of both the target and models were not reported.

**Figure 20:**
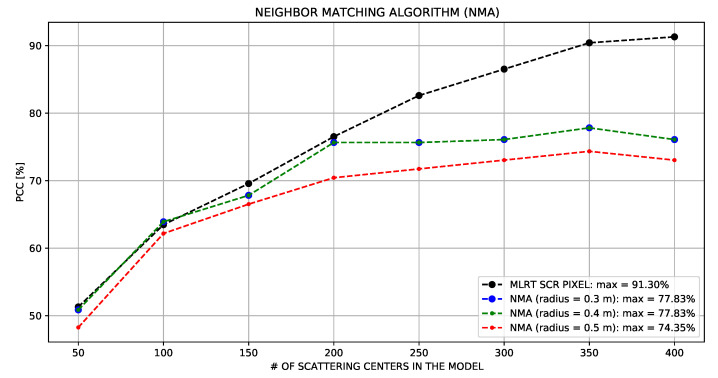
Comparison between the Neighbor Matching and the proposed algorithms.

For comparison purposes, we reproduced the experiment using NMA. Circumferences with the same dimensions as the original work were used. The number of scattering centers ranged from 50 to 400 with a step of 50. From analyzing the results presented in Figure 20, we saw that the algorithm increased the performance by increasing the number of scattering centers. However, increasing the number of scattering centers above 200 did not imply a PCC increase, which remained below 78%.

## 4. Discussion

The first experiment reported in this article compares different Goodness of Fit tests. As seen in Section 3.1.1, the LRT stood out regarding PXS and MSE variation. This result refers to the small samples due to a large number of categories (scattering centers). For small samples, the chi-square approximation in many cases did not fit well with the actual distribution [58]. In building the model, the expected value of each category had an average value of 1/N, where *N* was the number of scattering centers in the model. In an example with N=400, the average expected value was 0.0025, much less than 5, which is the minimum expected value required by PXS.

In Section 3.1.2, better accuracy in the results was observed when the hypotheses were generated based on the scattering centers of the test image (SC-based approach) compared to those obtained when the entire test image was used (SLC-based approach). In the SLC-based approach, when using the observed value of all scattering centers, the assumed risk was that the observed scattering centers would present random values as they tend to be very close to the noise level. As their amplitudes were very low, the randomness caused a substantial error to the categorical partial score. The SC-based approach addressed this problem by assigning zero to the observed value of scattering centers not found within the *N* ones extracted from the test image and then fixing a partial error of 100% for the category.

The goodness-of-fit test with weighting function was also efficient based on the intersection of the scattering center sets present in both the model and the test image. A greater coincidence of scattering centers was expected for the true class than the false classes. Therefore, assigning the number of elements at the intersection of the two sets of scattering centers as an inversely proportional weight further reduced the true hypothesis test score.

The simplification of the Point Spread Function (PSF) used by the CLEAN algorithm did not significantly improve accuracy. However, since it did not compromise the results, it is recommended for applications limited by processing resources and execution time.

It was observed that the SCR-based approach achieved the best performance among all implementations, indicating that the exclusion of low-ranking scattering centers from the target was worthy. The number of scattering centers to be excluded was approximated experimentally, showing that the PCC started to drop using a larger number of scattering centers. This result showed that there was an adequate number of scattering centers to represent the target properly, and more than that may have jeopardized the performance.

Lewis et al. [38], the developers of the SAMPLE dataset, provided not only measured and synthetic data but also some results of potential experiments and suggested performance metrics so that researchers could compare experimental results appropriately. In one of those experiments, the 10 classes were used for training and testing, varying the proportion of measured and synthetic data in the training batch. Using an algorithm based on a Convolutional Neural Network (CNN) with four convolutional layers and four fully connected layers, the average PCC was 24.97% when only synthetic data were used in the training batch.

Consideration must be made regarding a self-imposed constraint that potentially affected the accuracy of the reference experiments. Perhaps to maintain certain compatibility with previous works focused on the MSTAR dataset, thereby enabling a comparison of results, they used the 17-degree depression angle images exclusively for testing. The same condition was not applied in the proposed algorithm since its success was directly connected to the similarity between synthetic and measured data. Therefore, there was no reason to generate hypotheses based on models with different aspect angles. Furthermore, as the SAMPLE dataset lacked images at some aspect angles, the entire set was used to extract the maximum amount of images with the same aspect angles.

Lewis et al., in their most recent work, achieved average accuracies of 51.58% [39] and 82.05% [43] by training a DenseNet with the assistance of a Generative Adversarial Network (GAN). Scarnati et al. reached an accuracy of 55.62% [41] also by using DenseNet and no more than 35% [45] when using Complex-Valued Neural Networks (CVNN). To preprocess images and augment data with adversarial training, Inkawhich et al. used Deep Neural Networks (DNN) to achieve an average PCC of 95.06% [44]. Seller et al. [42] developed an algorithm based on CNN capable of achieving an accuracy of 95.1% when synthetic data correspond to 99% of the data used in training. However, when using 100% of synthetic data in training, the PCC dropped below 85%. Jennison et al. [47] achieved an accuracy of 88.45% although their synthetic data were transformed based on measured data, which in some ways can be considered a leak between test and training data. Finally, the best result obtained so far can be considered to be the one achieved by Melzer et al. [48]. They tested 53 different neural networks achieving an average PCC of 96.88% with a CNN VGG HAS.

**Figure 21:**
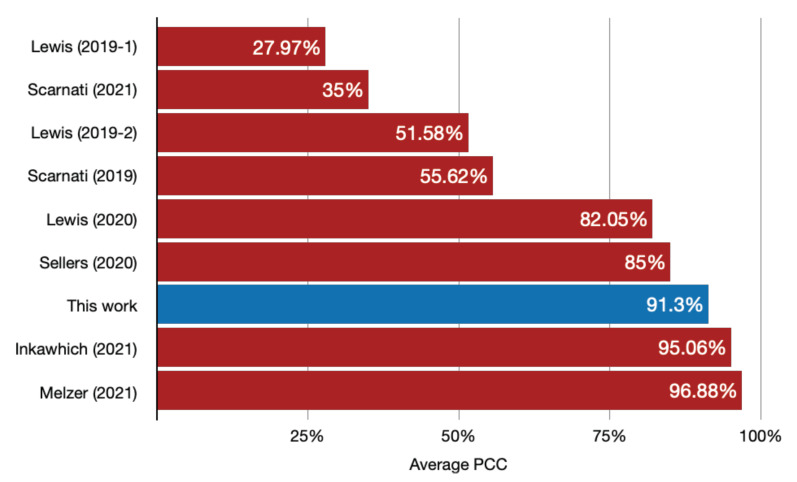
Classification results (PCC) of all works, the training of which was performed using purely synthetic data.

As can be seen in Figure 13e and Table 5, for the 10 classes the best SOC classification accuracy reached by our proposed algorithm was 91.30%. All classification results considering the SAMPLE dataset with purely synthetic data are summarized in Figure 21.

Although the proposed algorithm has been outperformed by two of the referenced works, its applicability can be recommended based on processing-speed requirements. All other works used machine learning/deep learning to train their algorithms. Although this work does not contemplate a study of processing speed, it is a fact that DNN and CNN require a high processing time for training. The proposed algorithm replace the training process with a direct data inspection method for building the model. During online classification, the proposed algorithm performed a single set of operations for a reduced number of scattering centers (400). The other algorithms performed, for all 16,384 image pixels, a large number of operations in each layer of their networks.

However, some constraints can characterize disadvantages in applying the proposed algorithm. The main one is dependence on the discriminator stage. If the discriminator stage cannot perfectly center the target on the chip and estimate its pose within the tolerance limits, the classification results will not be successful. Another drawback is the dependence on database completeness. To achieve operationally satisfactory results, it is necessary to simulate a wide range of target poses.

## 5. Conclusions

This article proposed a model-based classification algorithm addressing the SAMPLE dataset. As can be seen in Section 3.2, this work pioneered the SAMPLE dataset EOC classification relating to noise corruption and configuration variance. The algorithm proved to be robust and reliable when the added noise had an SNR of up to −5 dB. In the configuration variance experiment due to the particularities of the structures of the different serial numbers of the same class. In contrast to the SOC classification, the performance of the algorithm improved with a reduced number of scattering centers within the model.

Among several future works that may provide potential improvements to the proposed algorithm, we can highlight the generation of additional hypotheses. Despite the high agreement between the measured and simulated targets of the SAMPLE dataset, small variations in the scene and the acquisition geometry resulted in scattering centers migrating to neighboring cells, given the pixel spacing of only 20 cm. Therefore, for each hypothesis, eight additional hypotheses could be verified considering the possible migration of the target scattering centers set in each direction.

Using a secondary classification logic to decide on two or more hypotheses with close scores is also promising. If the difference between the score of the predicted class and the second-placed class does not exceed a threshold, the decision power can be transferred to a secondary algorithm. The secondary algorithm could explore different features not explored by the main algorithm, such as the physical dimensions of the target. Such an algorithm could be implemented using morphological transformations to obtain pixel clusters representative of the target dimensions.

## Figures and Tables

**Table 1:** SAMPLE dataset target classes (*k*).

*k*	0	1	2	3	4	5	6	7	8	9
Class	2S1	BMP-2	BTR-70	M1	M2	M35	M60	M548	T-72	ZSU-23

**Table 2:** Radar parameters.

$f_{c}$	*B*	Ω	$\theta_{c}$
9.6 GHz	591 MHz	$0.04^{\circ }$	$0^{\circ }$

**Table 3:** Aspect angles used in the experiments.

θ	ϕ
$16^{\circ }$	$14^{\circ }$, $16^{\circ }$, $17^{\circ }$, $18^{\circ }$, $21^{\circ }$, $23^{\circ }$, $29^{\circ }$, $31^{\circ }$, $35^{\circ }$, $36^{\circ }$, $37^{\circ }$, $38^{\circ }$, $41^{\circ }$, $44^{\circ }$, $58^{\circ }$
$17^{\circ }$	$14^{\circ }$, $15^{\circ }$, $18^{\circ }$, $19^{\circ }$, $32^{\circ }$, $37^{\circ }$, $38^{\circ }$, $51^{\circ }$

**Table 4:** Standard Operating Condition—Percentage of Correct Classification for different implementations resulting from 10-class within class classification tests.

	MSE	PXS	LRT	LRT	MLRT	MLRT	MLRT
	SLC	SLC	SLC	SC	SC	SC	SCR
	CLEAN	CLEAN	CLEAN	CLEAN	CLEAN	PIXEL	PIXEL
SC	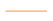	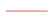	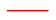	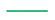	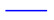	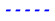	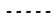
50	22.61%	24.35%	26.96%	49.13%	52.17%	49.13%	51.30%
100	29.57%	35.22%	37.89%	56.09%	57.83%	62.61%	63.48%
150	32.17%	37.83%	40.87%	63.91%	70.43%	65.65%	69.57%
200	33.91%	43.04%	45.22%	68.70%	74.78%	75.65%	76.52%
250	34.35%	47.39%	52.17%	70.43%	79.57%	79.57%	82.61%
300	37.39%	52.17%	54.35%	70.87%	82.17%	83.48%	86.52%
350	38.70%	54.78%	56.52%	74.78%	83.91%	86.96%	90.43%
400	39.13%	56.09%	59.57%	77.83%	85.22%	86.96%	91.30%

**Table 5:** Confusion matrix: Standard Operating condition (SOC), 400 Scattering Centers models, PIXEL extraction method, SCR-based approach, Modified Likelihood Ratio Test (MLRT), Global PCC = 91.30%.

Class	2S1	BMP-2	BTR-70	M1	M2	M35	M60	M548	T-72	ZSU-23	PCC
2S1	21	1	0	0	0	0	0	0	0	1	91.30%
BMP-2	2	21	0	0	0	0	0	0	0	0	91.30%
BTR-70	0	0	23	0	0	0	0	0	0	0	100%
M1	0	0	0	23	0	0	0	0	0	0	100%
M2	2	0	0	0	21	0	0	0	0	0	91.30%
M35	0	0	2	0	1	19	0	0	0	1	82.61%
M60	0	0	0	0	0	0	23	0	0	0	100%
548	0	0	0	0	0	0	0	19	0	4	82.61%
T-72	1	0	0	0	1	0	0	0	21	0	91.30%
ZSU-23	3	0	0	0	1	0	0	0	0	19	82.61%

**Table 6:** Extended Operating Condition—Percentage of Correct Classification (PCC) for different SNR resulting from noise contamination.

SC	SOC	SNR = +10	SNR = +5	SNR = 0	SNR = −5	SNR = −10
50	51.30%	48.70%	50.00%	49.57%	46.96%	48.26%
100	63.48%	64.78%	60.00%	61.30%	60.87%	52.61%
150	69.57%	66.52%	66.09%	66.09%	65.22%	58.26%
200	76.52%	76.09%	76.52%	76.52%	68.26%	64.78%
250	82.61%	82.17%	80.87%	79.57%	73.48%	64.35%
300	86.52%	83.48%	84.35%	81.74%	75.65%	65.65%
350	90.43%	88.26%	89.13%	85.65%	79.57%	68.26%
400	91.30%	90.00%	90.43%	89.13%	82.17%	68.70%

## Data Availability

The SAMPLE dataset was made obtained in accordance with the instructions contained in [38]. Available online: https://github.com/benjaminlewis-afrl/SAMPLE_dataset_public (accessed on 13 April 2021). The MSTAR dataset was obtained according to the U.S. Air Force Research Lab guidelines. Available on line: https://www.sdms.afrl.af.mil/index.php?collection=mstar (accessed on 13 April 2021).

## Abbreviations

The following abbreviations are used in this manuscript:

3D-CAD	Three Dimensional Computer Aided Design
ATR	Automatic Target Recognition
CNN	Convolutional Neural Networks
CVNN	Complex-Valued Neural Networks
DCT	Discrete Cosine Transform
DNN	Deep Neural Networks
EOC	Extended Operating Condition
EMC	Electromagnetic Computing
GAN	Generative Adversarial Network
GMM	Gaussian Mixture Model
GoF	Goodness of Fit Test
HLC	High Level Classifier
HMM	Hidden Markov Model
KNN	K-Nearest Neighbor
LDA	Linear Discriminant Analysis
LLC	Low Level Classifier
LRT	Likelihood Ratio Test
MLRT	Modified Likelihood Ratio Test
MSE	Mean Square Error Test
MSTAR	Moving and Stationary Target Acquisition and Recognition
NMA	Neighbor Matching Algorithm
NMF	Non-Negative Matrix Factorization
PCA	Principal Component Analysis
PCC	Percentage of Correct Classification
PSF	Point Spread Function
PXS	Pearson’s Chi-Square Test
SAMPLE	Synthetic and Measured Paired and Labeled Experiment
SAR	Synthetic Aperture Radar
SC	Scattering Center
SCR	Scattering Centers Reduction
SLC	Single Look Complex
SOC	Standard Operating Condition
SRC	Sparse Representation-based Classification
SVM	Support Vectors Machine
TM	Template Matching
WCG	Within Class Group

## References

[1] Moreira A., Prats-Iraola P., Younis M., Krieger G., Hajnsek I., Papathanassiou K.P. (2013). A tutorial on synthetic aperture radar. IEEE Geosci. Remote Sens. Mag..

[2] Cumming I.G., Wong F.H. (2005). Digital Processing of Synthetic Aperture Radar Data.

[3] Blacknell D., Vignaud L. ATR of ground targets: Fundamentals and key challenges. Radar Automatic Target Recognition (ATR) and Non-Cooperative Target Recognition (NCTR). 2013. https://www.sto.nato.int/publications/STO%20Educational%20Notes/STO-EN-SET-172-2013/EN-SET-172-2013-01.pdf.

[4] El-Darymli K., Gill E.W., McGuire P., Power D., Moloney C. (2016). Automatic target recognition in synthetic aperture radar imagery: A state-of-the-art review. IEEE Access.

[5] Jianxiong Z., Zhiguang S., Xiao C., Qiang F. (2011). Automatic target recognition of SAR images based on global scattering center model. IEEE Trans. Geosci. Remote Sens..

[6] Ding B., Wen G. (2018). A region matching approach based on 3-d scattering center model with application to SAR target recognition. IEEE Sens. J..

[7] Tan J., Fan X., Wang S., Ren Y. (2018). Target recognition of SAR images via matching attributed scattering centers with binary target region. Sensors.

[8] Zhou Z., Cao Z., Pi Y. (2019). Subdictionary-based joint sparse representation for SAR target recognition using multilevel reconstruction. IEEE Trans. Geosci. Remote Sens..

[9] Ding B., Wen G., Huang X., Ma C., Yang X. (2017). Data augmentation by multilevel reconstruction using attributed scattering center for SAR target recognition. IEEE Geosci. Remote Sens. Lett..

[10] Zhu X., Huang Z., Zhang Z. (2019). Automatic target recognition of synthetic aperture radar images via gaussian mixture modeling of target outlines. OPTIK.

[11] Zhang L., Zhang Y.H., Yin H.C., He S.Y., Zhu G.Q. (2019). A fast SAR target indexing method based on geometric models. IEEE Trans. Geosci. Remote Sens..

[12] Papson S., Narayanan R.M. (2012). Classification via the shadow region in SAR imagery. IEEE Trans. Aerosp. Electron. Syst..

[13] Gudnason J., Cui J., Brookes M. (2009). HRR automatic target recognition from superresolution scattering center features. IEEE Trans. Aerosp. Electron. Syst..

[14] Cui Z., Cao Z., Yang J., Feng J., Ren H. (2015). Target recognition in synthetic aperture radar images via non-negative matrix factorisation. IET Radar Sonar Navig..

[15] Dang S., Cui Z., Cao Z., Liu N. (2018). SAR target recognition via Incremental nonnegative matrix factorization. Remote Sens..

[16] Mishra A.K. Validation of PCA and LDA for SAR ATR. Proceedings of the TENCON 2008—2008 IEEE Region 10 Conference.

[17] Tian S., Lin Y., Gao W., Zhang H., Wang C. (2020). A multi-scale u-shaped convolution auto-encoder based on pyramid pooling module for object recognition in synthetic aperture radar images. Sensors.

[18] Lv J., Liu Y. (2019). Data augmentation based on attributed scattering centers to train robust CNN for SAR ATR. IEEE Access.

[19] Jones G., Bhanu B. (1999). Recognition of articulated and occluded objects. IEEE Trans. Pattern Anal. Mach. Intell..

[20] Bhanu B., Jones G. (2000). Recognizing target variants and articulations in synthetic aperture radar images. Opt. Eng..

[21] Bhanu B., Jones G. (2002). Increasing the discrimination of synthetic aperture radar recognition models. Opt. Eng..

[22] Bhanu B., Lin Y. (2003). Stochastic models for recognition of occluded targets. Pattern Recognit..

[23] Ding B., Wen G., Zhong J., Ma C., Yang X. (2016). Robust method for the matching of attributed scattering centers with application to synthetic aperture radar automatic target recognition. J. Appl. Remote Sens..

[24] Ma C., Wen G., Ding B., Zhong J., Yang X. (2016). Three-dimensional electromagnetic model-based scattering center matching method for synthetic aperture radar automatic target recognition by combining spatial and attributed information. J. Appl. Remote Sens..

[25] Jones G., Bhanu B. (2001). Recognizing articulated objects in SAR images. Pattern Recognit..

[26] Jones G., Bhanu B. (2001). Recognizing occluded objects in SAR images. IEEE Trans. Aerosp. Electron. Syst..

[27] Dungan K.E., Potter L.C. (2010). Classifying transformation-variant attributed point patterns. Pattern Recognit..

[28] Dungan K.E., Potter L.C. (2011). Classifying vehicles in wide-angle radar using pyramid match hashing. IEEE J. Sel. Top. Signal Process..

[29] Ding B., Wen G., Ma C., Yang X. (2018). An efficient and robust framework for SAR target recognition by hierarchically fusing global and local features. IEEE Trans. Image Process..

[30] Ding B., Wen G. (2019). Combination of global and local filters for robust SAR target recognition under various extended operating conditions. Inf. Sci..

[31] Ding B., Wen G., Zhong J., Ma C., Yang X. (2017). A robust similarity measure for attributed scattering center sets with application to SAR ATR. Neurocomputing.

[32] Ding B., Wen G., Huang X., Ma C., Yang X. (2017). Target recognition in synthetic aperture radar images via matching of attributed scattering centers. IEEE J. Sel. Top. Appl. Earth Obs. Remote Sens..

[33] Zhu J.W., Qiu X.L., Pan Z.X., Zhang Y.T., Lei B. (2017). An improved shape contexts based ship classification in SAR images. Remote Sensing.

[34] Ding B., Wen G. (2018). Target reconstruction Based on 3-d scattering center model for robust SAR ATR. IEEE Trans. Geosci. Remote Sens..

[35] Fan J., Tomas A. (2018). Target reconstruction based on attributed scattering centers with application to robust SAR ATR. Remote Sens..

[36] Jiang C., Zhou Y. (2018). Hierarchical fusion of convolutional neural networks and attributed scattering centers with application to robust SAR ATR. Remote Sens..

[37] Liu J., He S., Zhang L., Zhang Y., Zhu G., Yin H., Yan H. (2020). An Automatic and Forward Method to establish 3-d parametric scattering center models of complex targets for target recognition. IEEE Trans. Geosci. Remote Sens..

[38] Lewis B., Scarnati T., Sudkamp E., Nehrbass J., Rosencrantz S., Zelnio E., Zelnio E., Garber F.D. (2019). A SAR dataset for ATR development: The synthetic and measured paired labeled experiment (SAMPLE). Proceedings of the Algorithms for Synthetic Aperture Radar Imagery XXVI.

[39] Lewis B., DeGuchy O., Sebastian J., Kaminski J., Zelnio E., Garber F.D. (2019). Realistic SAR data augmentation using machine learning techniques. Proceedings of the Algorithms for Synthetic Aperture Radar Imagery XXVI.

[40] Paulson C.R., Nolan A.R., Goley G.S., Nehrbass S., Zelnio E., Zelnio E., Garber F.D. (2019). Articulation study for SAR ATR baseline algorithm. Proceedings of the Algorithms for Synthetic Aperture Radar Imagery XXVI.

[41] Scarnati T., Lewis B., Zelnio E., Garber F.D. (2019). A deep learning approach to the Synthetic and Measured Paired and Labeled Experiment (SAMPLE) challenge problem. Proceedings of the Algorithms for Synthetic Aperture Radar Imagery XXVI.

[42] Sellers S.R., Collins P.J., Jackson J.A. Augmenting simulations for SAR ATR neural network training. Proceedings of the 2020 IEEE International Radar Conference (RADAR).

[43] Lewis B., Cai K., Bullard C., Overman T.L., Hammoud R.I., Mahalanobis A. (2020). Adversarial training on SAR images. Proceedings of the Automatic Target Recognition XXX.

[44] Inkawhich N., Inkawhich M.J., Davis E.K., Majumder U.K., Tripp E., Capraro C., Chen Y. (2021). Bridging a gap in SAR-ATR: Training on fully synthetic and testing on measured data. IEEE J. Sel. Top. Appl. Earth Obs. Remote Sens..

[45] Scarnati T., Lewis B. Complex-valued neural networks for synthetic aperture radar image classification. Proceedings of the 2021 IEEE Radar Conference (RadarConf21).

[46] Inkawhich N.A., Davis E.K., Inkawhich M.J., Majumder U.K., Chen Y. (2021). Training SAR-ATR models for reliable operation in open-world environments. IEEE J. Sel. Top. Appl. Earth Obs. Remote Sens..

[47] Jennison A., Lewis B., DeLuna A., Garrett J., Zelnio E., Garber F.D. (2021). Convolutional and generative pairing for SAR cross-target transfer learning. Proceedings of the Algorithms for Synthetic Aperture Radar Imagery XXVIII.

[48] Melzer R., Severa W.M., Plagge M., Vineyard C.M., Overman T.L., Hammoud R.I., Mahalanobis A. (2021). Exploring characteristics of neural network architecture computation for enabling SAR ATR. Proceedings of the Automatic Target Recognition XXXI.

[49] Shan C., Huang B., Li M. (2018). Binary morphological filtering of dominant scattering area residues for SAR target recognition. Comput. Intell. Neurosci..

[50] Ross T.D., Worrell S.W., Velten V.J., Mossing J.C., Bryant M.L. (1998). Standard SAR ATR evaluation experiments using the MSTAR public release data set. Proceedings of the Algorithms for Synthetic Aperture Radar Imagery V.

[51] Kechagias-Stamatis O., Aouf N. (2021). Automatic target recognition on synthetic aperture radar imagery: A survey. IEEE Aerosp. Electron. Syst. Mag..

[52] Potter L., Moses R. (1997). Attributed scattering centers for SAR ATR. IEEE Trans. Image Process..

[53] Bhalla R., Ling H. (1996). Three-dimensional scattering center extraction using the shooting and bouncing ray technique. IEEE Trans. Antennas Propag..

[54] Segalovitz A., Frieden B. (1978). CLEAN-type deconvolution algorithm. Astron. Astrophys..

[55] Yun D.J., Lee J.I., Bae K.U., Yoo J.H., Kwon K.I., Myung N.H. (2017). Improvement in computation time of 3-d scattering center extraction using the shooting and bouncing ray technique. IEEE Trans. Antennas Propag..

[56] Doerry A.W. (2017). Catalog of Window Taper Functions for Sidelobe Control.

[57] Ozdemir C. (2012). Inverse Synthetic Aperture Radar Imaging with MATLAB Algorithms.

[58] Larntz K. (1973). Small Sample Comparison of Likelihood-Ratio and Pearson Chi-Square Statistics for the Null Distribution.

